# Decoding bronchopulmonary dysplasia in premature infants through an epigenetic lens

**DOI:** 10.3389/fmed.2025.1531169

**Published:** 2025-04-03

**Authors:** Seyed Alireza Dastgheib, Reza Bahrami, Mohammad Golshan-Tafti, Mahsa Danaei, Sepideh Azizi, Amirhossein Shahbazi, Maryam Yeganegi, Amirmasoud Shiri, Ali Masoudi, Hossein Neamatzadeh

**Affiliations:** ^1^Department of Medical Genetics, School of Medicine, Shiraz University of Medical Sciences, Shiraz, Iran; ^2^Neonatal Research Center, Shiraz University of Medical Sciences, Shiraz, Iran; ^3^Department of Pediatrics, Islamic Azad University of Yazd, Yazd, Iran; ^4^Department of Obstetrics and Gynecology, School of Medicine, Iran University of Medical Sciences, Tehran, Iran; ^5^Shahid Akbarabadi Clinical Research Development Unit, Iran University of Medical Sciences, Tehran, Iran; ^6^School of Medicine, Ilam University of Medical Sciences, Ilam, Iran; ^7^Department of Obstetrics and Gynecology, School of Medicine, Iranshahr University of Medical Sciences, Iranshahr, Iran; ^8^School of Medicine, Shiraz University of Medical Sciences, Shiraz, Iran; ^9^School of Medicine, Shahid Sadoughi University of Medical Sciences, Yazd, Iran; ^10^Mother and Newborn Health Research Center, Shahid Sadoughi University of Medical Sciences, Yazd, Iran

**Keywords:** epigenetics, bronchopulmonary dysplasia, DNA methylation, RUNX3, microRNAs, long non-coding RNAs

## Abstract

This review provides a comprehensive overview of the evolving insights into the epigenetic mechanisms associated with bronchopulmonary dysplasia (BPD). It specifically highlights the roles of DNA methylation, histone modifications, and RNA regulation in the development of BPD in premature infants. BPD results from complex interactions among genetic factors, environmental exposures, and neonatal stressors. Key findings suggest that intrauterine hypoxia, hyperoxia, and nutrition can lead to epigenetic alterations, affecting gene expression and methylation, which may serve as biomarkers for early BPD detection. RUNX3 is identified as a critical transcription factor influencing lung development and inflammation, while changes in DNA methylation and histone dynamics in cord blood are linked to immune dysregulation associated with BPD. The role of m6A RNA methylation regulators from the IGF2BP family affects mRNA stability and gene expression relevant to BPD. Additionally, specific histones and microRNAs, particularly from the miR-17∼92 cluster, are implicated in pulmonary development and vascular regulation. Long non-coding RNAs (lncRNAs), such as MALAT1, also play a role in gene regulation via competitive endogenous RNA networks, indicating their potential as biomarkers and therapeutic targets. The interplay of these epigenetic mechanisms underscores the need for further research to develop targeted interventions aimed at reducing BPD severity and enhancing health outcomes for at-risk neonates.

## Introduction

Bronchopulmonary dysplasia (BPD) is a chronic lung disease predominantly affecting premature infants, resulting from inadequate lung development often associated with mechanical ventilation and supplemental oxygen use (1, 2). These interventions can induce inflammation and scarring, particularly harming the alveoli, which are crucial for gas exchange (3). BPD primarily impacts infants born before 28 weeks of gestation who require respiratory support, with high-pressure ventilation and elevated oxygen levels exacerbating the condition. Symptoms of BPD include rapid or labored breathing, shortness of breath, apnea, wheezing, and cyanosis, which indicates low blood oxygen levels (4). Diagnosis typically relies on the necessity for supplemental oxygen after 28 days of life or upon reaching 36 weeks of postmenstrual age (PMA), often supplemented by chest X-rays and blood tests (5, 6). The severity of BPD is classified as mild, moderate, or severe based on the level of respiratory support required and the infant’s overall health, which guides treatment decisions and predicts long-term outcomes (7).

Epigenetics, a rapidly advancing field, examines how non-genetic factors influence gene expression without altering the DNA sequence, emphasizing the complex interplay between genetic predispositions and environmental factors, particularly in relation to diseases (8, 9). In the context of BPD, both genetic and environmental influences encountered before and after birth significantly contribute to its development (2, 7). Adverse prenatal conditions, such as intrauterine hypoxia, hyperoxia, and maternal smoking, have been associated with lasting changes in gene expression through epigenetic modifications, potentially increasing the risk of BPD and affecting future generations. Additionally, psychosocial stress may alter the epigenetic landscape, potentially accelerating biological aging and heightening the risk of developing BPD (10). Ongoing research into these epigenetic changes holds promise for improving perinatal health strategies and facilitating more personalized medical and public health interventions (11). Notable findings include alterations in DNA methylation linked to critical pathways involved in lung maturation, hematopoiesis, inflammation, and cellular mechanisms in infants predisposed to BPD (12).

Recent studies employing epigenome-wide association studies (EWAS) have identified a substantial number of differentially methylated CpG sites, with 275 sites exhibiting differential methylation at a false discovery rate of less than 1%. Remarkably, approximately 64% of these CpGs were hypomethylated in BPD cases compared to controls. Among these, the CpG site cg23328237, situated in the 3’ untranslated region (UTR) of an unidentified gene, demonstrated a particularly strong association with BPD. The differentially methylated loci corresponded to 386 nearby genes, highlighting the extensive implications of methylation changes on gene expression pertinent to lung development and BPD pathology (12). Moreover, studies revealed that higher nucleated red blood cell (NRBC) content in preterm cord blood significantly influenced DNA methylation profiles. Elevated NRBC percentages correlated with lower birth weight (BW) and gestational age (GA), as well as hypomethylation of markers associated with tobacco smoke exposure (12, 13). Transcriptomic analyses indicated that gene expression changes in cord blood cells were reflective of cell cycle regulation, developmental processes, and pulmonary disorders related to BPD. Additionally, intrauterine hypoxia was found to elicit epigenetic changes, including altered DNA methylation, histone acetylation, and variations in miRNA expression (10).

Current research highlights the potential of specific epigenetic biomarkers as predictors of BPD risk, emphasizing the need for further studies to validate these findings and explore epigenetic therapies for prevention and treatment. This review aims to clarify the complex relationship between BPD and epigenetic mechanisms, focusing on how epigenetic modifications may influence the pathophysiology of this chronic lung condition. By synthesizing existing literature, we highlight the role of various epigenetic factors—such as DNA methylation, histone modification, and non-coding RNAs—in modulating inflammatory and fibrotic responses in the lungs of preterm infants. We also assess the clinical implications of these changes for early diagnosis and the development of targeted therapeutic strategies. The manuscript is structured to first discuss research progress on epigenetic mechanisms in BPD, followed by an examination of the epigenetic regulation of immune responses, epigenome-wide association studies, and the influence of environmental factors. It further explores the role of RUNX3, cord blood epigenetics, m6A methylation, histone modifications, microRNA dysregulation, long non-coding RNAs, competitive endogenous RNA networks, sex differences, and DNA methylation in animal models, concluding with a discussion on DNA methylation clocks for assessing health outcomes in preterm infants. Ultimately, this review aspires to deepen our understanding of BPD from an epigenetic perspective and to guide future research initiatives.

## Materials and methods

This review aims to synthesize existing literature on BPD in premature infants, emphasizing epigenetic factors. A systematic approach was employed to gather relevant studies from multiple databases, including PubMed, Scopus, Web of Science, Google Scholar, Embase, Cochrane Library, CINAHL, and PsycINFO, using keywords such as “bronchopulmonary dysplasia,” “premature infants,” “epigenetics,” “DNA methylation,” “histone modification,” “RNA regulation,” “genetic factors,” “environmental exposures,” “neonatal stressors,” “intrauterine hypoxia,” “hyperoxia,” “nutrition,” “RUNX3,” “immune dysregulation,” “m6A RNA methylation,” “IGF2BP,” “microRNAs,” “MALAT1,” “therapeutic targets,” and “non-coding RNA.” Studies published up to November 2024 were included, focusing on peer-reviewed articles, reviews, and clinical studies that explore epigenetic mechanisms influencing BPD in preterm infants, with only English-language studies considered. Relevant information was extracted from selected articles, including study design, sample size, demographic data, and key findings related to epigenetic mechanisms, paying special attention to methodologies like genomic analyses and methylation profiling. The extracted data were categorized based on various epigenetic mechanisms, such as DNA methylation patterns, histone modifications, and the role of non-coding RNAs, allowing for an analysis of patterns and correlations between epigenetic changes and BPD severity. A narrative synthesis integrated findings from diverse studies, highlighting the interplay between genetic susceptibility, environmental factors, and epigenetic modifications in BPD pathophysiology. Gaps in the current literature were identified, and recommendations for future research directions were formulated. Since this review involved synthesizing existing literature rather than direct research with human subjects or animals, ethical approval was not required, although adherence to ethical standards in research reporting was maintained. The outcomes of this systematic review aim to contribute to a deeper understanding of the epigenetic underpinnings of BPD in premature infants and may inform future therapeutic strategies.

## Research progress in the epigenetic mechanisms of BPD

**FIGURE 1:**
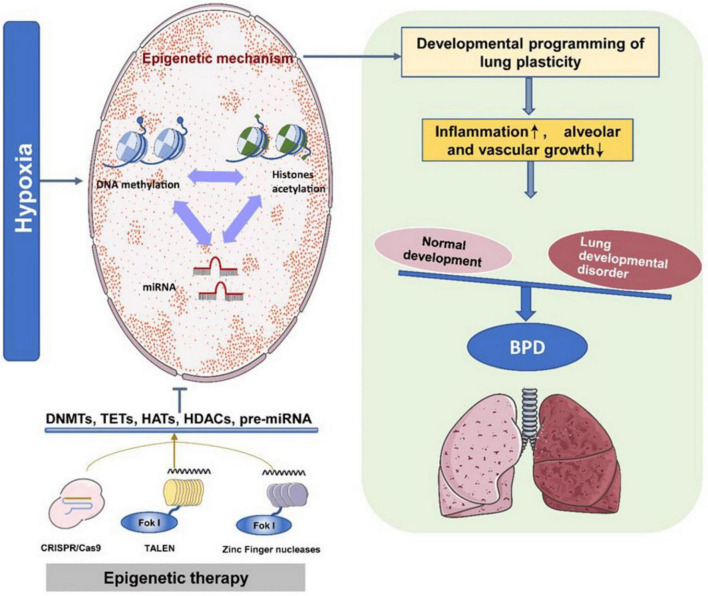
The epigenetic program regulates bronchopulmonary dysplasia (BPD) development. Intrauterine hypoxia triggers various epigenetic mechanisms that coordinate lung plasticity. This results in inflammation during lung development, inhibiting the growth of alveoli and blood vessels. Consequently, the balance between normal and abnormal lung development is disrupted, leading to BPD. Epigenetic therapies utilizing DNMT inhibitors, histone deacetylase (HDAC) inhibitors, and miR modulators can enhance lung development and mitigate the onset of neonatal chronic lung disease. This figure is derived from a study by Tong et al. (10).

Bronchopulmonary dysplasia poses a substantial risk to premature infants, manifesting as inadequate lung development with potential long-term consequences (14). The disorder arises from a complex interplay of genetic predisposition, environmental exposures, and prenatal and postnatal risk factors (15). Epigenetic mechanisms, especially DNA methylation, have emerged as key regulators of gene expression in the context of BPD (16). Alterations in gene expression and DNA methylation during lung development suggest that epigenetic changes may play a critical role in BPD’s pathogenesis (16, 17). Factors such as intrauterine hypoxia, hyperoxia, and disturbances in chromatin remodeling pathways have been linked to BPD development (18, 19). Intrauterine hypoxia triggers epigenetic mechanisms that affect lung plasticity, leading to inflammation during lung development and impeding the growth of alveoli and blood vessels. This disruption results in an imbalance in lung development, contributing to BPD. Epigenetic therapies, such as DNMT inhibitors, HDAC inhibitors, and miR modulators, can promote lung development and lower the risk of neonatal chronic lung disease (Figure 1). Environmental influences, particularly hypoxia and hyperoxia, have been shown to impact epigenetic programming within lung development and BPD (19, 20). For instance, hyperoxia-induced methylation changes, including reduced expression of the RUNX3 gene, have been documented in a rat model of BPD, suggesting a connection between epigenetic modifications and disease development (19). Further, disruptions in gene expression related to chromatin remodeling pathways have been observed in premature infants at risk for BPD, implying a crucial role for epigenetic dysregulation in susceptibility to the disorder (18). EWAS have revealed DNA methylation loci affiliated with BPD, underscoring the significance of epigenetic changes in the disease’s development (21). Additionally, variations in the expression of microRNAs and their relationship with DNA methylation patterns have been noted in severe cases of BPD, further emphasizing the role of epigenetic regulation in determining disease severity (22). Moreover, differences in DNA methylation among very preterm infants have been linked to serious neonatal morbidities, including BPD, highlighting the influence of these epigenetic changes on health outcomes (23). The regulation of the immune system has also been shown to be affected by epigenetic modifications in experimental models of BPD, suggesting a connection between epigenetics and immune responses regarding the disorder (24). Furthermore, analyses of the epigenome and transcriptome of cord and peripheral blood from preterm infants at risk for BPD have provided valuable insights into the epigenetic factors underlying susceptibility (21). Table 1 presents critical insights into the epigenetic mechanisms and their implications BPD. Together, these findings illustrate a complex interplay between epigenetic modifications, gene expression, and immune responses in the pathogenesis of BPD.

**TABLE 1:** Essential insights into epigenetic mechanisms and their implications for bronchopulmonary dysplasia (BPD).

Section	Key points
RUNX3 role	– RUNX3 is critical for lung development and BPD regulation. – Disruption in RUNX3 expression is linked to abnormal lung architecture and impaired alveolarization. – RUNX3 may serve as a prognostic marker and therapeutic target for BPD. – DNA methylation and H3K27me3 alterations affect RUNX3 in BPD models. – Increased DNMT1 and DNMT3b expression correlates with decreased RUNX3 levels in hyperoxia models.
Cord blood modifications	– Methylation alterations impact immune responses in premature neonates. – Epigenetic markers in cord blood DNA are associated with BPD risk. – Studies show differential patterns of hypomethylation and hypermethylation in BPD patients. – Specific genes (e.g., CTSH, SPOCK2) linked to BPD identified through epigenome-wide association studies. – Elevated neutrophil-to-lymphocyte ratio (NLR) may indicate higher BPD risk.
m6A methylation	– m6A modification regulates mRNA metabolism and gene expression. – IGF2BP proteins are involved in BPD pathogenesis, promoting mRNA stability. – YTHDF2 reduces mRNA stability, affecting hematopoietic stem cell proliferation. – METTL3 enhances hyperoxia-induced pyroptosis in BPD. – Down-regulation of several m6A regulators is observed in BPD cohorts.
Histone modifications	– Histone acetylation/deacetylation regulates gene transcription. – Changes in histone modification patterns (e.g., hyperacetylation of H2A.Z and H3K9) are linked to BPD. – Histone deacetylase 3 (HDAC3) is pivotal in abnormal pulmonary angiogenesis and alveolar development. – Targeting histone acetylation and chromatin remodeling may offer therapeutic avenues.
MicroRNA dysregulation	– Specific microRNAs (e.g., miR-21, miR-34a, miR-431) are differentially expressed in BPD. – miR-17∼92 cluster downregulation correlates with BPD severity. – Elevated levels of miR-421 target FGF10, exacerbating inflammation and apoptosis in BPD. – miR-29b administration may enhance lung phenotype in severe BPD models.
Long non-coding RNAs	– lncRNAs play roles in transcription, RNA metabolism, and chromatin modification. – Differential expression of lncRNAs (e.g., MALAT1, lncRNA_AK096792) is observed in BPD. – lncRNA_AK096792 may serve as a biomarker for BPD.
Competitive endogenous RNA networks	– ceRNA networks regulate gene expression in BPD. – Dysregulated ceRNA networks may serve as therapeutic targets and biomarkers.
Animal model studies	– Animal models help explore epigenetics of BPD. – Sex-specific differences in epigenetic responses to hyperoxia are noted. – DNA methylation may hinder alveolarization in neonatal rats.
Neonatal epigenetic clocks	– NEOage clocks assess biological maturity and predict health outcomes in preterm infants. – Initial findings link epigenetic clocks to BPD development and risk assessment.

## Epigenetic regulation of immune responses in BPD

Epigenetic regulation is a pivotal factor in modulating immune responses associated with BPD in preterm infants. It encompasses mechanisms such as DNA methylation, histone modifications, and non-coding RNAs that together influence gene expression and immune function (25). Studies indicate that preterm infants exhibit DMRs in their placentas and cord blood compared to full-term infants, which align with genes that play significant roles in immune regulation and inflammation. The altered DNA methylation patterns are believed to exacerbate the heightened inflammatory responses characteristic of BPD, particularly due to increased methylation of inflammation-related genes that sustain inflammatory processes central to BPD pathogenesis (26). In addition, variations in histone acetylation and methylation can modify chromatin structure, thereby affecting gene expression tied to immune responses and lung development by altering the accessibility of transcription factors. Non-coding RNAs, particularly microRNAs, also play a crucial role by post-transcriptionally regulating gene expression and influencing the differentiation and activation pathways of immune cells (27). Moreover, m6A RNA methylation is instrumental in modulating immune responses related to BPD, with changes in m6A methylation regulators, such as IGF2BP1/2/3, linked to the disease and its immune environment (28, 29). These epigenetic alterations impact immune cell composition and signaling pathways, highlighting the significance of DNA and histone modifications during lung development and injury.

In the context of BPD, immune dysregulation creates an imbalance between pro-inflammatory and anti-inflammatory signals. Preterm infants frequently display increased levels of regulatory T cells (Tregs) in early life, potentially acting as a protective mechanism before the onset of BPD (30). While Tregs initially contribute to mitigating inflammation, their efficacy may be undermined in the inflammatory milieu of BPD, causing them to adopt a more inflammatory phenotype that could exacerbate lung injury. Additionally, oxidative stress stemming from hyperoxia can inflict inflammatory damage on alveolar epithelial cells, further worsening the inflammatory state in BPD. Changes in gut microbiota and blood transcriptomes are also associated with immune dysregulation in preterm infants, compounding this chronic lung condition (31). The polarization and activation of macrophages are essential processes, with DNA methylation influencing gene expression throughout lung development. Understanding the interplay between epigenetics and immune function is vital for developing targeted therapeutic strategies to address environmental factors that induce epigenetic modifications. This relationship highlights the potential for interventions targeting specific epigenetic alterations to improve immune function and reduce the risk of BPD in preterm infants (32, 33). The adaptability of immune cells, particularly Tregs, suggests a protective role against excessive immune activation; however, their responses may vary significantly based on the surrounding activation environment. Continued research is essential to explore these epigenetic mechanisms and their clinical applications in preventing or alleviating BPD in vulnerable populations.

## Epigenome-wide association studies in BPD

Epigenome-wide association studies have revealed the epigenetic mechanisms behind BPD, highlighting the role of epigenetic modifications in understanding the disease’s etiology and identifying potential biomarkers for early detection. Cuna et al. (16) investigated the effects of DNA methylation on gene expression during lung alveolar septation in murine and human models. They identified 95 genes in mice that showed an inverse relationship between expression and methylation during normal septation, focusing on genes vital for lung development, particularly those associated with Wnt signaling and the extracellular matrix. In human samples, 23 genes demonstrated differential methylation and reciprocal expression changes in BPD compared to preterm and term lung tissues, particularly involving detoxifying enzymes and TGF-β signaling. Importantly, 20 genes and three pathways were common to both murine and human studies, emphasizing DNA methylation’s key role in regulating gene expression linked to normal and abnormal alveolar septation (16). Wang et al. (12) studied the connections between GA, BW, and blood cell composition in premature infants with BPD. Analyzing cord blood DNA from 14 BPD-affected preterm infants and 93 unaffected ones, they found significant associations with GA (*p* < 1.0E-04) and BW (*p* < 1.0E-02). A negative correlation was noted between nucleated red blood cell (NRBC) percentage and both BW and GA, with NRBC-rich samples exhibiting a hypomethylation profile associated with tobacco exposure. They identified 38 differentially methylated CpGs tied to pathways involved in lung maturation and hematopoiesis, and observed an increased epigenetic mutation burden in infants who developed BPD (adjusted *p* = 0.02). While the sample size was small, transcriptomic changes in cord blood highlighted critical biological processes related to lung development and cell proliferation (12). In a 2020 study, Everson et al. (23) sought to identify PMA-associated CpGs while minimizing the detection of surrogate markers during buccal swab collection. Their EWAS revealed that infants with more health complications had longer NICU stays, which influenced DNA methylation. They identified significant epigenetic signals with varying associated CpGs at different significance thresholds, incorporating PMA as a covariate for some CpGs. A separate analysis identified ten genome-wide significant CpGs, three of which were intergenic, with cg09787236 on 6q13 deemed the most significant. Notably, cg26838315 (10q21.1) exhibited lower DNA methylation levels with increased PMA, and sensitivity analyses indicated that adjustments for immune cell proportions did not affect the results. Analysis of differentially methylated regions (DMRs) identified 1,744 candidate regions, none of which reached Bonferroni-adjusted significance; however, seven DMRs had regional *p*-values within the FDR 10% threshold (23). Comparative analyses of existing EWAS enhance the understanding of the epigenetic landscape related to BPD, revealing strong connections between specific DNA methylation patterns and the risk of developing the condition. This underscores the urgent need for further research into these biomarkers for early detection and intervention, suggesting that integrating epigenetic and transcriptomic data could inspire innovative therapeutic strategies to mitigate BPD’s impact on vulnerable populations.

## Role of environmental factors in the epigenome of BPD

Environmental factors significantly affect the epigenome of preterm infants, influencing their risk of BPD through various mechanisms. Preterm infants often face challenges such as underdeveloped lungs due to lower GA, leading to epigenetic changes that hinder lung maturation. Low BW can also cause detrimental modifications that impact lung function and immune development (4, 12). Exposure to neonatal stressors like mechanical ventilation and oxygen therapy disrupts DNA methylation in immune-related genes, resulting in chronic inflammation and impaired lung growth (34). Intrauterine hypoxia induces epigenetic alterations, including changes in DNA methylation and histone acetylation, which compromise alveolarization and increase BPD risk (10). Practices in neonatal intensive care units can further contribute to lung injury and epigenomic changes by downregulating genes related to mitochondrial biogenesis. Additionally, variations in the airway microbiome influence immune development and modify the epigenetic landscape, potentially worsening BPD. Recent studies, such as those by Wang et al. (12) and Cho et al. (21), highlight the impact of environmental factors on the epigenome, revealing differential methylation of inflammatory genes crucial for lung development and linking epigenetic changes to transcriptomic signatures in immune responses. Maternal stress, nutrition, and pollutant exposure can disrupt DNA methylation, as noted by Song and Bhandari (35), while glucocorticoid exposure during critical development stages has been shown to alter DNA methylation, increasing BPD susceptibility. Understanding the interactions among gestational conditions, inflammatory responses, and environmental exposures is essential for identifying biomarkers for early detection and developing strategies to mitigate BPD severity, ultimately improving health outcomes for vulnerable infants (35). Recent research has explored the connection between air pollution and epigenetic markers associated with BPD in preterm infants, indicating that exposure to pollutants like nitrogen dioxide (NO2) and particulate matter (PM) can alter DNA methylation patterns. Long-term NO2 exposure is linked to significant global hypomethylation in gene bodies and shores of CpG islands, crucial for regulating genes involved in lung development and inflammation. Studies on prenatal exposure have revealed both global and locus-specific DNA methylation changes in placental and cord blood samples, with higher levels of ozone (O3) correlating with reduced methylation in neonates (36). A review indicated that air pollution impacts DNA methylation throughout the lifespan, suggesting that early-life exposure significantly influences respiratory health and elevates BPD risk. While the mechanisms by which air pollution modifies the epigenome are still being studied, these changes are believed to sensitize the immune system, increasing the likelihood of inflammatory conditions like BPD (37). Understanding these pathways is vital for developing targeted interventions and identifying potential biomarkers for early BPD risk associated with environmental pollutants, underscoring the necessity for future research to focus on specific environmental exposures and their epigenetic effects to enhance therapeutic approaches.

Maternal smoking during pregnancy is a significant risk factor for BPD in infants, particularly those born prematurely who may require supplemental oxygen and respiratory support. A systematic review of 171,772 infants revealed a strong association between maternal smoking and an increased risk of moderate to severe BPD, with a pooled risk ratio of 1.126. However, no significant correlation was found for all BPD cases or severe BPD specifically (38). The harmful chemicals in tobacco smoke, particularly nicotine, can cross the placental barrier and hinder fetal lung development. Animal studies indicate that maternal smoking results in increased oxidative stress and inflammation in offspring’s lungs, leading to structural changes that heighten the risk of respiratory problems (39). Moreover, smoking during pregnancy is associated with preterm birth, another known BPD risk factor, underscoring the importance of smoking cessation for better neonatal outcomes. Tobacco smoke can also induce epigenetic changes in offspring, affecting DNA methylation, microRNA expression, and histone modifications, which may disrupt lung development-related gene expression, such as that of the AHRR and CYP1A11 genes (38, 40). These epigenetic alterations can interfere with crucial developmental pathways and contribute to long-term health issues, potentially increasing the risk of respiratory diseases like asthma and chronic obstructive pulmonary disease (COPD) (41).

## RUNX3’s role in BPD development

The Runt-related transcription factor 3 (RUNX3) is crucial for lung development, especially in the differentiation of lung epithelial cells and the establishment of pulmonary vasculature (42). The subcellular localization of RUNX3 is vital for its functional activity; when RUNX3 is localized in the cytoplasm, it has been associated with tumorigenesis (43). RUNX3 modulates gene expression through post-transcriptional mechanisms that involve DNA methylation at the 5′-terminal and transcription start sites (44). This process is primarily mediated by DNA methyltransferases (DNMTs), with a particular emphasis on DNMT1 and DNMT3b (45). Furthermore, the tri-methylation of lysine 27 on histone H3 (H3K27me3), driven by EZH2, contributes significantly to this regulatory framework (46). The expression levels of RUNX3 play a crucial role in determining its effectiveness, while H3K27me3 trimethylation represents a common epigenetic alteration linked to its activity (47).

RUNX3’s role in the development of BPD highlights its regulatory capacity in lung maturation and inflammatory responses (25). Research indicates that the expression of RUNX3 is frequently altered in BPD, which may result in abnormal lung structure and impaired alveolar development. In particular, diminished levels of RUNX3 in lung tissue from patients with BPD may induce epithelial-mesenchymal transition (EMT) in alveolar type II (AT2) cells, negatively impacting alveolar growth (48). Furthermore, studies involving mouse models of asthma have shown that the mislocalization of Runx3 protein is associated with allergic inflammation and increased airway hyper-responsiveness (49). These findings suggest that RUNX3 could be utilized as both a prognostic biomarker and a potential therapeutic target for BPD. Recent research has identified a correlation between DNA methylation and alterations in H3K27me3 in the promoter region of the RUNX3 gene within a hyperoxia-induced neonatal mouse model of BPD. Specifically, RUNX3 protein is prominently expressed in bronchioles and alveolar epithelial cells on embryonic day 17.5 throughout the pulmonary tissue of mice. Investigations into the cellular mechanisms suggest that epigenetic modifications of the RUNX3 gene significantly impact BPD development. Given the established role of DNA methylation in this condition, it is hypothesized that decreased expression of essential pulmonary development genes, such as RUNX3, may be linked to these epigenetic alterations (50). Zhu et al. (19) explored the relationship between RUNX3 protein levels and DNMT expression in alveolar type 2 (AT2) cells, reporting a marked increase in DNMT1 protein expression after 1 day of hyperoxia exposure, with DNMT3b levels rising notably from day ten onward and inversely correlating with RUNX3 protein levels. While both control and experimental AT2 cell groups showed elevated methylation, the hyperoxia group exhibited significantly higher methylation levels, particularly noticeable from day fourteen. Consequently, hypermethylation of the RUNX3 promoter in AT2 cells from the hyperoxia model group was evident compared to control cells. This study emphasizes the interplay between DNA methylation and H3K27me3 in a rat model of hyperoxia-induced BPD, highlighting the combined effects of DNMT3b-mediated DNA methylation and EZH2-mediated H3K27me3 on the downregulation of RUNX3 protein during the later stages of BPD. Early identification and intervention targeting these epigenetic pathways may alleviate the impacts of environmental and genetic factors on premature infants, thereby reducing the risk of developing BPD (19). Additionally, the findings underscore the significance of Runx3 in the pathophysiology of BPD in neonatal rat models and its relationship with EMT under hyperoxic conditions. A notable decrease in Runx3 protein and mRNA in BPD-derived alveolar type 2 cells correlates with critical pulmonary development markers, implying that low Runx3 expression may promote EMT, thereby hindering alveolar maturation. Experimental evidence indicates that Runx3 knockdown in RLE-6TN cells under TGF-β1 stimulation initiates EMT, whereas its overexpression inhibits this process, establishing Runx3 as a vital regulator of EMT dynamics. Furthermore, in a hyperoxia model, the significant downregulation of RUNX3, alongside the upregulation of DNMT3b and EZH2, along with epigenetic modifications, indicates a complex regulatory mechanism influencing RUNX3 expression. Treatments with JMJD3 and DZNep effectively reversed RUNX3’s hyperoxia-induced downregulation, suggesting potential therapeutic strategies to alleviate BPD by targeting Runx3’s epigenetic regulation and related pathways (19, 50). A better understanding of the molecular mechanisms governing RUNX3’s role in BPD may lead to the discovery of novel therapeutic targets for this complex condition.

## Impact of cord blood epigenetics on BPD in preterm infants

Methylation alterations and transcriptional dysregulation are believed to have impacted neutrophil and lymphocyte levels, as well as T cell and adaptive immune responses, in premature neonates. These changes may also have influenced inflammation, phagocytosis, cellular assembly, and DNA damage repair (51, 52). Modifications in methylation and disrupted transcription could have affected the ratios of neutrophils and lymphocytes and the functions of T cells and adaptive immune responses. Such modifications likely played a role in various biological processes relevant to premature neonates, including inflammation, phagocytosis, cellular assembly, and DNA damage repair (53, 54). A methylation-based framework has indicated a positive correlation between reduced BW and GA with increased levels of nucleated red blood cells (NRBCs). However, variability exists, as some larger and more mature infants present high levels of NRBCs. Highlighting the significance of GA in neonatal health, researchers have investigated integrating cord blood DNA methylation data into predictive models for GA. This integration has led to the identification of epigenetic gestational age (EGA) acceleration, which reflects the difference between GA estimated from DNA methylation (epigenetic maturity, EGA) and GA determined through ultrasound or last menstrual period (chronological GA) (55–57).

**FIGURE 2:**
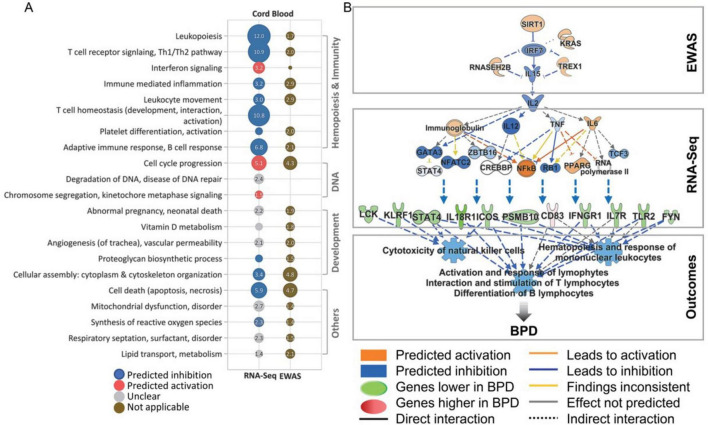
Overview of cord blood genes and pathways linked to bronchopulmonary dysplasia (BPD) and a comparison of epigenetic changes. **(A)** The dot plot illustrates enriched pathways and gene ontology (GO) terms from differentially expressed genes in BPD cases versus non-BPD controls, with pathway analysis adjusted for covariates like cell type proportions, sex, gestational age, and birth weight. Pathways related to cord blood epigenome-wide association studies (EWAS) are highlighted for their relevance to BPD. Circle size represents pathway or GO significance, while color indicates activation trends. **(B)** Ingenuity Pathway Analysis (IPA) identifies interleukin 2 (IL-2) as a crucial factor influencing epigenomic and transcriptomic changes in blood cell development and function during BPD pathogenesis. Key molecules in this network—SIRT1, TREX1, and IRF2—play roles in regulating cytokine signaling and transcription, potentially impacting BPD progression. This figure is derived from a study by Cho et al. (21).

As shown in Figure 2, The analysis of cord blood genes and pathways associated with BPD reveals significant insights into the condition’s underlying mechanisms, particularly when considering epigenetic modifications. A comprehensive examination of differentially expressed genes in BPD cases, when compared to non-BPD controls, highlights enriched pathways and Gene Ontology (GO) terms, as visually represented in a dot plot. This pathway analysis has been rigorously adjusted for various covariates, including cell type proportions, sex, gestational age, and birth weight. Notably, pathways identified through cord blood epigenome-wide association analysis (EWAS) are underscored for their relevance to BPD. Furthermore, utilizing Ingenuity Pathway Analysis (IPA), the interleukin-2 (IL-2) pathway emerges as a crucial modulator of both epigenomic and transcriptomic changes in blood cell development and function, which are instrumental in the pathogenesis of BPD. Key molecules within this network, including SIRT1, TREX1, and IRF2, play significant roles in regulating cytokine signaling and transcriptional processes, thereby potentially influencing the progression and severity of BPD. This multifaceted approach underscores the intricate interplay of genetic and epigenetic factors in BPD’s development and progression, highlighting potential avenues for therapeutic intervention (21).

In a detailed examination involving 54 preterm infants, Cohen et al. (18) discovered changes in histone acetyltransferase binding activity along with pathways linked to chromatin remodeling. While the individual genes analyzed did not exhibit statistical significance within the cord blood samples, the associated pathways did yield significant findings. In a study conducted by Cho et al. (21) on preterm infants, epigenetic markers in cord blood DNA associated with the risk of BPD were identified. These markers were linked to specific genes such as cathepsin H (CTSH, cg24847366) and SPOCK2 (cg17958658). The study proposed that alterations in the cord blood methylome, related to processes such as lung and tissue development, cell cycle regulation, leukopoiesis, immune-mediated inflammation, and T and B cell responses, could serve as indicators for the potential development of BPD. Furthermore, EWAS on cord blood samples have revealed differential patterns of hypomethylation and hypermethylation of CpG islands in patients diagnosed with BPD, including alterations in the SPOCK2 gene and genes involved in the pathway of reactive oxygen species production (21). Similarly, Cho et al. (21) investigated DNA methylation and gene expression in cord and venous blood within the first month following premature birth, unveiling molecular and cellular differences in newborns later diagnosed with BPD. A notable correlation was observed between GA and reduced lymphocyte proportions, paired with increased granulocyte proportions in both cord and peripheral blood samples. These variations were also reflected in the neutrophil-to-lymphocyte ratio (NLR), particularly in extremely premature newborns who subsequently developed BPD. Elevated NLR values in the early weeks of life may signal a greater risk for subsequent BPD development, corroborating findings from previous research that indicated higher NLR values in infants with BPD at each time point assessed (21). In 2022, Wang et al. conducted an analysis of DNA methylation in cord blood to investigate the relationship between GA, BW, and the distribution of cord blood cell types, specifically NRBCs, in a small cohort of preterm infants with and without BPD. The study noted no significant differences in the methylation-based estimated proportions of various cell types within cord blood DNA between infants with BPD and their counterparts without the condition. Furthermore, no differences in cell-type composition were observed among non-BPD infants who did not require supplemental oxygen. Their results demonstrated that the composition of cord blood cells, inferred from methylation patterns, varied with BW and GA, establishing that higher levels of NRBCs were associated with lower BW and DNA hypomethylation (12).

## m6A methylation in gene regulation related to BPD

N6-methyladenosine (m6A) is recognized as the predominant internal modification present in messenger RNA (mRNA) within eukaryotic cells. This modification plays a pivotal role in regulating multiple facets of mRNA metabolism, including splicing, stability, localization, and translation. The dynamic and reversible characteristics of m6A modification enable precise control over gene expression in response to various cellular signals and environmental stimuli (58, 59). m6A is implicated in numerous biological processes such as neuronal development, tumorigenesis, gametogenesis, and both physiological and pathological events. Importantly, m6A modification is involved in mRNA quality control, guiding improperly processed mRNAs for nuclear retention and degradation (59, 60). The regulation of m6A is mediated by “writers” (for example, METTL3, METTL14, RBM15, WTAP, KIAA1429), “erasers” (such as ALKBH5 and FTO), and “readers” (including IGF2BPs and YTHDFs). Overall, m6A modification is a significant posttranscriptional alteration that influences various biological functions (59, 61). The advent of adenosine deamination sequencing (AD-seq) has enabled the detection of m6A in RNA with single-base resolution, offering critical insights into the roles of m6A in cellular processes. Recent research has illuminated the involvement of m6A modification in the development and progression of various diseases (62, 63). However, the precise mechanisms through which m6A affects BPD remain incompletely understood.

Modifications in m6A RNA methylation regulators, particularly IGF2BP1/2/3, have been observed in BPD. Analyses of differentially expressed genes underscore the significant influence of IGF2BP in BPD pathogenesis. The IGF2BP family of m6A reader proteins (IGF2BP1/2/3) targets mRNA transcripts by recognizing the m6A consensus motifs “GGAC,” promoting the stability, storage, and translation of certain mRNA targets. The absence of IGF2BPs is associated with widespread decreases in the regulation of target genes (64). Functional enrichment analysis of IGF2BP-targeted genes reveals their significant roles in DNA replication, the cell cycle, cell proliferation, and cancer. Unlike IGF2BP1/2/3, the m6A reader YTHDF2 decreases the stability of target mRNAs, promoting their degradation (65). YTHDF2 inhibits the Wnt signaling pathway, crucial for cellular communication and influencing development, cell proliferation, and differentiation. It achieves this by binding to and degrading mRNAs of key genes such as ccnd1, c-Myc, and Axin2, which reduces the proliferation and differentiation of hematopoietic stem cells. As an m6A “writer,” METTL3 is vital for mRNA stability, preferentially binding to m6A-modified RNAs, typically near stop codons and in 3’-UTRs. This modification is associated with various biological functions and pathological events, with high METTL3 expression linked to poor survival in lung cancer patients. Furthermore, METTL3-mediated m6A modification of GPX4 and STAT2 promotes ferroptosis in conditions like NET-induced sepsis-associated acute lung injury and neonatal pneumonia (59). In a 2023 study, Xu et al. (66) revealed that METTL3 enhances hyperoxia-induced pyroptosis in BPD by inhibiting the LC3-conjugation pathway, shedding new light on BPD development. Additionally, the FTO “eraser” reduces m6A methylation in human cells through the demethylation of m6A. Suppressing FTO activity and elevating m6A levels fosters the recruitment of YTHDF1, leading to the increased translation of MYC and thereby contributing to tumorigenesis (66). According to Bao et al. (91), demonstrated that the expression of several m6A regulators—namely YTHDF1, YTHDF2, ZC3H13, FTO, ELAVL1, LRPPRC, RBM15B, METTL14, CBLL1, and FMR1—was down-regulated in the BPD cohort compared to controls, whereas the levels of IGF2BP1, IGF2BP2, and IGF2BP3 were found to be elevated (64).

## Histone modifications and their role in BPD pathogenesis

The pathogenesis of BPD is closely associated with histone modifications, particularly the dynamic processes of acetylation and deacetylation. Histone acetylation generally activates gene transcription, while deacetylation represses it (67). Notable changes in histone modification patterns, such as hyperacetylation of H2A.Z and H3K9, have been observed at gene loci implicated in BPD, including NOS3 and STAT3. These alterations may contribute to the dysregulated vascular responses seen in children diagnosed with BPD (68).

Histone acetyltransferases (HATs) facilitate the addition of acetyl groups to lysine residues on histones, which neutralizes their positive charge, reducing their affinity for DNA and leading to a more relaxed chromatin structure that promotes gene activation (69). Research by Chao et al. (68) examining mice in a hyperoxic environment—used to model BPD—demonstrated elevated expression levels of NOS3 and STAT3 mRNA in lung endothelial cells, alongside changes in histone acetylation at the H2A.Z and H3K9 loci. In vitro cell culture experiments further supported the notion that histone acetylation at these loci is a significant factor in BPD development in premature infants (70). Notably, infants born before 28 weeks of gestation exhibit two epigenetic pathways associated with BPD—histone acetylation and chromatin remodeling. Targeting these pathways may offer promising therapeutic avenues (12).

Histone deacetylation, mediated by histone deacetylases (HDACs), results in the silencing of transcription by promoting tight nucleosome binding to DNA. Class I HDACs, such as HDAC1 and HDAC2, are primarily located in the nucleus and play essential roles in embryonic development, cellular proliferation, and differentiation (71). HDAC3 is particularly important in abnormal pulmonary angiogenesis and alveolar development associated with BPD, activating specific pathways that accelerate abnormal lung blood vessel and air sac growth (72). Reduced levels and activity of HDACs due to preterm birth or environmental stress contribute to lung development issues in BPD (48). Enhancing HDAC activity may provide a protective effect against BPD through inflammation reduction. Additionally, sirtuin-3 (Sirt3), another NAD-dependent deacetylase, helps protect lung cells from BPD-related damage by influencing FOXO1 activity. Studies using chorioamnionitis models, where lipopolysaccharides (LPS) were injected into the amniotic cavity of pregnant mice, revealed obstructed lung development similar to BPD phenotypes. Evaluating lung tissue from these newborns highlighted significant reductions in HDAC1 and HDAC2 expression compared to controls, suggesting these enzymes’ protective roles in alveolar development (73, 74). A related study examined Silent Information Regulator 1 (Sirt1) through tracheal aspirate fluid samples from 51 infants, finding lower Sirt1 levels in those with BPD, indicating a significant link between histone deacetylation and BPD progression (75). Londhe et al. (76) proposed that reduced expressions of HDAC1 and HDAC2 can lead to alveolar dysplasia, particularly in hyperoxic conditions. Systemic sepsis has been shown to trigger acute inflammatory responses in developing lungs, potentially leading to phenotypes resembling BPD in premature infants. Evidence suggests that HDAC inhibitors may worsen sepsis, further influencing inflammatory responses and impairing lung development, pointing to a significant role for these inhibitors in BPD etiology (76). These findings indicate that histone modifications are crucial regulators in the development and treatment of BPD, offering insights into the molecular mechanisms involved in lung development and dysplasia.

## MicroRNA dysregulation in BPD

Several microRNAs, including miRNA-21, miRNA-34a, miRNA-431, Let-7f, and miRNA-335, have been identified as differentially expressed in lung tissues affected by BPD. These microRNAs provide valuable insights into the underlying mechanisms contributing to BPD (77). Notably, microRNAs are implicated in critical processes such as branching morphogenesis and secondary septation, both essential for lung development and alveolarization. Additionally, specific microRNA signatures have been detected in the tracheal aspirates of preterm infants with severe BPD, suggesting their potential as biomarkers for assessing disease severity (78). The miR-17∼92 cluster, which is transcribed as a single unit, undergoes posttranscriptional modification to produce six distinct miRNAs: miR-17, miR-18a, miR-19a, miR-19b, miR-20a, and miR-92. These miRNAs exhibit high expression levels during lung development and have been shown to activate the EZH1-p65-Pgf axis through the inhibition of miR-17, leading to abnormal pulmonary angiogenesis and impaired alveolarization in BPD mouse models (79).

Research indicates that approximately 20 miRNAs are up-regulated and 26 are down-regulated in the alveolar compartment during BPD, highlighting their significant role in the disease’s onset and progression. In a pivotal study conducted by Rogers et al. (79) in 2015, it was found that all components of the miR-17∼92 cluster were downregulated in infants who succumbed to BPD as compared to those who died from other causes. The study further reported increased methylation in the promoter region of this cluster, associated with elevated expression of DNMTs (DNMT-1, 3a, and 3b) (79). Another investigation by the same group revealed substantial promoter methylation within the lung miR-17∼92 cluster in a severe BPD model. While control mice exhibited approximately 2% promoter methylation, mice subjected to LPS/O2 conditions presented with an alarming 98% methylation rate. Importantly, the research established circulating plasma miR-17 levels as an early indicator of disease severity, detectable just 5 days post-birth, prior to the onset of clinical symptoms (22). These findings point toward a model where alterations in miR-17∼92 cluster expression are driven by increased promoter methylation mediated by DNMTs. In addition to miR-17∼92, the work of Lal et al. (81) revealed that decreased expression of miR-876-3p at birth can effectively predict severe BPD in very low BW infants. Enhancing miR-876-3p activity has been linked to improvements in abnormal alveolar structures, suggesting its potential as a predictive biomarker for preventing BPD in this vulnerable population (80, 81). Further research by Sun et al. utilized microarray technology to analyze peripheral blood from neonates with BPD and a control group, identifying significantly elevated levels of serum miR-495-5p in the BPD group. This miRNA was found to target 117 genes involved in processes such as apoptosis, cell death, autophagy, transcriptional regulation, and angiogenesis, indicating its possible role in regulating BPD development (82).

Clinical studies have corroborated the reduced expression of miR-29b in the serum of premature infants diagnosed with BPD. Animal studies indicated that administering miR-29b could enhance lung phenotype in severe BPD models, hinting at its potential as a new therapeutic avenue for the prevention or treatment of severe BPD. In hyperoxia-induced BPD mouse models, high-throughput sequencing has identified 201 differentially expressed miRNAs, including miR-342 and miR-335, with notable down-regulation of miR-150, miR-126, and miR-151, while miR-21 and miR-34a were found to be up-regulated (83, 84). The expression of miR-30a significantly increased in neonatal mice lung tissue after prolonged hyperoxia exposure, demonstrating a gender difference, as female mice showed higher levels than male mice. Additionally, the expression level of miR-34a in the lung tissues of newborn mice exposed to hyperoxia has been shown to significantly increase; inhibiting miR-34a expression has been demonstrated to ameliorate BPD symptoms and associated pulmonary hypertension (85, 86). Conversely, overexpression of miR-34a exacerbates these symptoms, suggesting that miR-34a inhibitors could serve as potential therapeutic agents for BPD management (87). MiR-421 targets fibroblast growth factor 10 (FGF10). In hyperoxia-induced BPD models, researchers observed elevated miR-421 levels and reduced FGF10 levels in lung tissues. This dysregulation worsens inflammation and increases cell apoptosis in BPD lung tissue, suggesting that down-regulating miR-421 could be a potential therapeutic strategy to alleviate its harmful effects in BPD pathology (88).

## Long non-coding RNAs in BPD

Long non-coding RNAs (lncRNAs), which are defined as non-coding RNAs longer than 200 bases, represent about 80% of all ncRNAs. These molecules are crucial in various biological processes, including transcription, translation, RNA metabolism, chromatin modification, stem cell maintenance and differentiation, autophagy, apoptosis, and embryonic development. Due to their significant links with various diseases, lncRNAs have become key targets in research aimed at understanding disease mechanisms, thereby providing insights for treatment and prevention strategies (89). lncRNAs serve both as scaffolds for chromatin modification complexes and as direct transcription regulators. For example, some antisense lncRNAs bind to the 3’ UTR of mRNA, affecting its stability and interaction with microRNAs, while also performing various nuclear functions (90). A landmark study by Bao et al. first documented variations in lncRNA expression in lung tissues of mice exposed to hyperoxia. Specifically, they found that in the BPD group, 882 lncRNAs were up-regulated and 887 down-regulated, suggesting a potential role in BPD’s onset and progression and paving the way for a better understanding of its molecular mechanisms (91).

In a follow-up analysis of the GSE25286 dataset from the Gene Expression Omnibus (GEO), researchers examined the expression of the 8,778-base pair lncRNA, Metastasis Associated Lung Adenocarcinoma Transcript 1 (MALAT1), in the lung tissue of BPD mice. The results showed significant up-regulation of MALAT1 in the BPD group, with peripheral blood samples from premature infants also indicating increased MALAT1 levels in those affected by BPD. These findings suggest a close association between MALAT1 expression and the onset and progression of BPD, offering valuable clinical insights (92). Further research in China compared lncRNA_AK096792 levels in umbilical cord blood from premature infants with those in peripheral venous blood from neonates diagnosed with BPD. Results revealed significantly higher levels of lncRNA_AK096792 in the umbilical cord blood of the BPD group compared to the non-BPD group, with even higher levels in the peripheral blood of children with BPD than in umbilical samples, indicating its potential as a BPD biomarker. In another study, Cheng et al. (94) established a neonatal mouse model of BPD using hyperoxia and utilized Illumina sequencing to analyze lncRNA expression differences between affected and control groups. Their analysis identified 30,225 genes in the hyperoxia group and 30,361 lncRNA-related gene expressions in controls, revealing significant variations in lncRNA expression profiles. Among 1,175 different lncRNAs identified, 544 were up-regulated and 631 down-regulated (93). Gene Ontology (GO) enrichment analysis revealed 673 functional enrichment differences primarily related to biological processes such as cell positioning. Moreover, KEGG enrichment analysis indicated lncRNA involvement in 257 KEGG pathways, with nine lncRNAs validated experimentally. The significant differences in validated lncRNAs between the hyperoxia and control groups led researchers to propose that lncRNAs contribute to BPD development, thus presenting new perspectives for exploring the biological processes underlying the condition (94).

## Competitive endogenous RNA networks in BPD

Competitive endogenous RNA (ceRNA) networks are essential regulatory systems in which non-coding RNAs (ncRNAs) and messenger RNAs (mRNAs) compete for shared microRNA (miRNA) binding, thereby influencing gene expression and various biological processes and diseases. In BPD, studies highlight the significance of lncRNA-mediated ceRNA networks in regulating GTPase activity, ERK1 and ERK2 signaling pathways, chromosome regulation, and cell cycle control in mouse models (90). Extracellular signal-regulated kinases 1 and 2 (ERK1/2) are key components of the mitogen-activated protein kinase (MAPK) signaling pathway, which is critical for cellular processes such as proliferation, differentiation, and survival. This pathway is activated by stimuli like growth factors, cytokines, and oncogenes, involving a series of phosphorylation events that transmit signals from the cell membrane to the nucleus (95). Dysregulated ceRNA networks have also been associated with lung cancer, particularly lung adenocarcinoma, affecting multiple biological functions and offering prognostic and diagnostic potential. Studies, including those by Li et al. (96) have identified several novel lncRNAs as promising biomarkers and therapeutic targets for BPD, while Dong et al. (97) highlighted specific regulatory axes like miR17hg-miR-130b-3p-Robo2 and GM20455-miR-34a-5p-Brinp1. This research has enhanced our understanding of the molecular mechanisms underlying BPD, which mainly affects premature infants. The ceRNA hypothesis suggests that various RNA molecules—lncRNAs, miRNAs, and mRNAs—interact through shared miRNA response elements (MREs), with lncRNAs and circular RNAs (circRNAs) acting as “sponges” that sequester miRNAs, thereby regulating target mRNA expression.

Recent studies identified 445 differentially expressed genes and 155 differentially expressed miRNAs in neonates with BPD compared to healthy controls, enabling the construction of ceRNA networks that highlight specific lncRNAs crucial for regulating miRNA activity in BPD. Functional validation through quantitative real-time PCR (qPCR) in animal models has confirmed the biological relevance of these interactions, improving our understanding of lung development and injury mechanisms in neonates (98). This knowledge clarifies the pathogenesis of BPD and suggests potential therapeutic strategies, indicating that targeting specific lncRNAs or miRNAs within these regulatory networks could effectively mitigate lung injury and improve outcomes for affected infants. Notably, lncRNAs in the ceRNA framework act as miRNA “sponges,” preventing miRNAs from binding to target mRNAs, resulting in increased gene expression, while miRNAs can also enhance gene expression by competing with lncRNAs. Dysregulated lncRNA-miRNA interactions have been linked to BPD, with studies showing that in a mouse model, the ceRNA axis involving miR17hg, miR-130b-3p, and the roundabout guidance receptor 2 (Robo2) was disrupted, leading to the upregulation of miR17hg and Robo2 and downregulation of miR-130b-3p (99). Similarly, another regulatory axis involving GM20455, miR-34a-5p, and BMP/retinoic acid-inducible neural specific 1 (Brinp1) exhibited dysregulation, with GM20455 and Brinp1 upregulated and miR-34a-5p downregulated. Additionally, an analysis of circRNAs in BPD identified three upregulated circRNAs—hsa_circ_0007054, hsa_circ_0057950, and hsa_circ_0120151—that contribute to immune dysregulation and inflammatory responses through their interactions with miRNAs (30, 100). These findings indicate that dysregulated lncRNA-miRNA interactions significantly contribute to BPD pathogenesis, particularly affecting immune responses, inflammation, and lung development, highlighting the potential for targeting specific ceRNA axes as a novel therapeutic strategy against BPD.

## The role of Sex differences and DNA methylation in animal models

Ethical concerns have been raised regarding the practice of drawing blood from premature infants for research, primarily due to the invasive nature of the procedure and the complications associated with repeated use of cannulas. To address these challenges, researchers are encouraged to explore alternative methodologies, such as animal models, which can provide valuable insights into the epigenetics of BPD and enhance our understanding of its biological impacts (101). Animal models have proven particularly useful in examining sex-specific epigenetic variations following exposure to hyperoxia, as clinical outcomes for BPD often differ based on biological sex (67). Studies involving male and female mice have shown distinct patterns of histone acetylation in genes critical for lung development, highlighting the importance of sex as a variable in BPD research. For instance, chromatin immunoprecipitation targeting the H3K27ac histone modification has demonstrated significant sex-related differences in the epigenetic response to hyperoxia. Specifically, female mice exhibit an upregulation of miR-30a, a microRNA that targets genes linked to angiogenesis (102). This finding is significant as it indicates that female mice exhibit more substantial changes in angiogenesis-related gene expression, particularly in the Delta-like 4 (Dll4) gene, which is essential for the Notch signaling pathway. These mechanisms may help maintain pulmonary vascular development in females after exposure to high oxygen levels compared to males (103). This signaling pathway is a conserved intercellular mechanism vital for various biological processes, including embryonic development, cell fate determination, and tissue homeostasis in multicellular organisms (104).

Recent animal studies have begun to illuminate the role of DNA methylation modifications in the development of BPD. For instance, research by Chen et al. has drawn parallels between the lung phenotype observed in hyperoxic lung injury models and the underlying pathophysiology of BPD. This investigation employed DNA methylation co-immunoprecipitation techniques to analyze whole-genome DNA methylation profiles in rat lung tissue, revealing that DNA methylation may hinder alveolarization processes induced by hyperoxia in neonatal rats (94). Complementary work by Bik-Multanowski et al. (105) utilized microarray technology to explore methylation levels in the lung tissue of neonatal rat models of BPD resulting from hyperoxic exposure. Their findings demonstrated increased DNA methylation levels in the promoter regions of key genes, such as transforming growth factor beta receptor 1 (TGFBR1) and cyclic adenylate response element binding protein 1 (CREB1), indicating a potential role for DNA methylation in the pathogenesis of BPD (105). Further research has demonstrated that hyperoxic conditions can profoundly affect the phosphatidylinositol-3-kinase (PI3K)-protein kinase B (AKT) signaling pathway in mouse models of BPD. Notable changes in the expression of genes associated with BPD, along with hypermethylation of key components within this signaling pathway, strengthen the hypothesis that epigenetic modifications play a critical role in the regulatory mechanisms underlying BPD development (74, 106). The PI3K-AKT signaling pathway is essential for regulating numerous cellular processes, including growth, survival, proliferation, and metabolism (107). Overall, these findings emphasize the necessity of utilizing animal models to dissect the intricate epigenetic landscape associated with BPD, drawing attention to both sex-specific differences and the impact of DNA methylation pathways.

## DNA methylation clocks for assessing preterm health outcomes

DNA methylation-based epigenetic clocks, or neonatal aging epigenetic clocks (NEOage clocks), show promise in evaluating biological maturity and predicting health outcomes for preterm infants. These clocks align with chronological age, allowing for an assessment of neonatal development related to both immediate and long-term health issues (108, 109). Recent research links NEOage clocks to the development of BPD, demonstrating their effectiveness in estimating clinical GA and identifying epigenetic biomarkers early associated with BPD. Studies emphasize DNA methylation as a biomarker for biological age and disease progression in preterm infants, highlighting specific CpG site patterns that expose the gap between biological and chronological ages (110). NEOage clocks provide better predictions of PMA and postnatal age (PNA) in very preterm infants compared to conventional methods. Evidence suggests that accelerated biological aging, as indicated by these clocks, correlates with increased risks of neonatal morbidities, including moderate to severe BPD. However, the variability in DNA methylation during early development complicates accurate measurement. Integrating NEOage clocks into clinical practice could improve risk assessment for preterm infants, though factors such as developmental differences and population characteristics may influence their accuracy (111). Ongoing research seeks to refine these clocks and address clinical implementation challenges. In 2021, Graw et al. (109) introduced four NEOage clocks specifically for estimating PMA and PNA, analyzing DNA methylation at certain CpG sites in very preterm infants. The Neonatal Neurobehavior and Outcomes in Very Preterm Infants (NOVI) study collected buccal cell samples from 542 infants to evaluate DNA methylation levels. The resulting NEOage clocks demonstrated strong correlations with actual ages and were compatible with Illumina EPIC and 450K arrays (109). Further research in 2023 by Paniagua et al. (111) found that infants with neonatal morbidities, especially BPD, exhibited signs of accelerated epigenetic aging. While some neurobehavioral traits showed varying levels of age acceleration, most did not significantly correlate with early-life age acceleration. A lower GA at birth appears to be a key factor influencing these associations. Although additional studies are necessary to clarify the relationship between NEOage clocks and BPD, initial findings are promising for managing this common neonatal lung condition (111).

## Potential applications of epigenetic findings in clinical practice

The exploration of epigenetic mechanisms in BPD has yielded significant insights applicable to clinical practice, particularly in developing diagnostic biomarkers and personalized therapies to improve outcomes for preterm infants. Specific epigenetic markers, such as DNA methylation patterns and altered microRNA levels, hold promise for creating diagnostic tools that could facilitate early identification of at-risk infants through routine screenings. This integration of epigenetic data into clinical assessments allows for targeted monitoring and interventions, potentially reducing the incidence and severity of BPD. Furthermore, understanding individual variability in epigenetic modifications can lead to personalized treatment strategies, enhancing therapy effectiveness through tailored interventions such as DNMT inhibitors, HDAC inhibitors, and microRNA modulators. Insights from epigenetic research can also inform targeted interventions addressing specific risk factors, such as promoting smoking cessation programs and reducing exposure to environmental pollutants during pregnancy. Additionally, epigenetic markers may serve as indicators for monitoring disease progression and treatment response, enabling healthcare providers to adjust care plans accordingly. The influence of maternal nutrition and environmental conditions on epigenetic programming underscores the importance of tailored nutritional support and optimized care environments. Integrating epigenetic findings into clinical protocols can enhance the standard of care for preterm infants, while collaboration among clinicians, geneticists, and researchers can lead to comprehensive care plans that consider both clinical and environmental factors in BPD management. Overall, the application of epigenetic findings in clinical practice represents a significant advancement in the prevention and management of BPD, contributing to the evolution of precision medicine in neonatology.

## Limitations

Research on the epigenetic mechanisms underlying BPD offers both advantages and limitations. A significant advantage is the potential to identify novel biomarkers and therapeutic targets, enhancing our understanding of the complex interactions between genetics, environmental factors, and nutrition in BPD development. Insights from EWAS could facilitate early detection and intervention, ultimately improving health outcomes for premature infants. The focus on environmental influences underscores the need for better care practices in neonatal intensive care units and preventive measures against pollution and maternal malnutrition. However, limitations include relatively small sample sizes in some studies, which may affect the generalizability of findings, and the dynamic nature of epigenetic modifications that can vary over time and with different exposures. Establishing causal relationships between epigenetic changes and BPD remains a significant challenge, necessitating further research to validate biomarkers for clinical use. The exploration of specific factors such as RUNX3, histone modifications, microRNA dysregulation, and lncRNAs highlights their potential as prognostic biomarkers and therapeutic targets, though complexities in gene regulation and reliance on animal models may complicate the translation of findings to human populations. Additionally, the study of sex differences and DNA methylation through animal models provides controlled experimental insights but may not fully replicate human conditions, emphasizing the need for continued research to bridge the gap between experimental results and clinical applications. Overall, while promising avenues for understanding and treating BPD exist, further studies are essential to address these challenges and translate findings into effective clinical practices.

## Implications and future directions

Research on epigenetic mechanisms in BPD holds significant implications for healthcare, particularly in enhancing clinical practices for managing at-risk premature infants. The findings from this study could lead to improved management strategies and highlight the necessity for further exploration of these mechanisms, paving the way for future research on long-term outcomes and potential therapies. By identifying specific DNA methylation patterns associated with BPD, opportunities arise for early detection and monitoring, enabling healthcare providers to recognize at-risk infants through routine screenings and implement timely interventions. Future studies should prioritize longitudinal research to assess the stability and predictive value of identified epigenetic biomarkers over time, evaluate their effectiveness in predicting BPD risk and outcomes, and explore the interplay between genetic predispositions and environmental factors to gain deeper insights into epigenetic modifications and their long-term effects on lung development. Investigating the impact of various therapeutic interventions on epigenetic changes in preterm infants with BPD is crucial for developing personalized treatment strategies. Additionally, examining the influence of maternal health, nutrition, and lifestyle on the epigenetic profiles of both mothers and infants could inform preventative measures and interventions. Integrating multi-omics approaches—combining genomics, transcriptomics, and epigenomics—could provide a comprehensive understanding of the biological mechanisms underlying BPD, ultimately guiding future research and clinical practice. Addressing environmental factors, such as maternal smoking and air pollution, during prenatal care is vital for reducing BPD risks, while reassessing neonatal care practices in light of stressors like mechanical ventilation is essential for optimizing outcomes. Monitoring inflammatory responses is also crucial, as elevated markers can indicate BPD progression and prompt timely interventions. Continued research into therapeutic targets, including long non-coding RNAs and microRNAs, is critical for developing effective treatments and biomarkers. A multidisciplinary approach involving neonatologists, geneticists, and public health experts is necessary to comprehensively address the complexities of BPD. By integrating epigenetic insights into clinical protocols, care can be enhanced, while ethical considerations in research emphasize the importance of safe methodologies that prioritize the well-being of vulnerable populations. Overall, these insights underscore the significance of a holistic patient management approach that considers both biological and chronological age to improve outcomes for preterm infants affected by BPD.

## Conclusion

In summary, the intricate interplay of genetic and epigenetic factors, including the roles of RUNX3, histone modifications, microRNA dysregulation, lncRNAs, and DNA methylation, is crucial in understanding the pathogenesis of BPD in premature infants. Research has revealed that these epigenetic mechanisms significantly influence lung development, immune responses, and inflammatory processes associated with BPD. RUNX3 has emerged as a potential prognostic biomarker and therapeutic target, while epigenetic modifications in cord blood DNA can help identify at-risk infants for preventive strategies. The dynamic processes of histone acetylation, the role of microRNAs, and the interactions between lncRNAs and microRNAs further underscore the importance of the epigenetic landscape in BPD. Additionally, advancements like NEOage clocks for assessing biological age through DNA methylation hold promise for predicting health outcomes. Together, these insights underscore the need for a comprehensive understanding of BPD’s etiology and the potential for innovative therapeutic strategies that could improve the health trajectories of vulnerable neonatal populations.
